# Cell Clearing Systems Bridging Neuro-Immunity and Synaptic Plasticity

**DOI:** 10.3390/ijms20092197

**Published:** 2019-05-04

**Authors:** Fiona Limanaqi, Francesca Biagioni, Carla Letizia Busceti, Larisa Ryskalin, Paola Soldani, Alessandro Frati, Francesco Fornai

**Affiliations:** 1 Human Anatomy, Department of Translational Research and New Technologies in Medicine and Surgery, University of Pisa, Via Roma 55, 56126 Pisa (PI), Italy; f.limanaqi@studenti.unipi.it (F.L.); larisa.ryskalin@unipi.it (L.R.); paola.soldani@dmu.unipi.it (P.S.); 2 I.R.C.C.S Neuromed, Via Atinense, 86077 Pozzilli (IS), Italy; francesca.biagioni@neuromed.it (F.B.); carla.busceti@neuromed.it (C.L.B.); alessandro.frati@uniroma1.it (A.F.)

**Keywords:** autophagy, proteasome, immunoproteasome, mTOR, T-cells, glia, dopamine, glutamate, neuro-inflammation

## Abstract

In recent years, functional interconnections emerged between synaptic transmission, inflammatory/immune mediators, and central nervous system (CNS) (patho)-physiology. Such interconnections rose up to a level that involves synaptic plasticity, both concerning its molecular mechanisms and the clinical outcomes related to its behavioral abnormalities. Within this context, synaptic plasticity, apart from being modulated by classic CNS molecules, is strongly affected by the immune system, and vice versa. This is not surprising, given the common molecular pathways that operate at the cross-road between the CNS and immune system. When searching for a common pathway bridging neuro-immune and synaptic dysregulations, the two major cell-clearing cell clearing systems, namely the ubiquitin proteasome system (UPS) and autophagy, take center stage. In fact, just like is happening for the turnover of key proteins involved in neurotransmitter release, antigen processing within both peripheral and CNS-resident antigen presenting cells is carried out by UPS and autophagy. Recent evidence unravelling the functional cross-talk between the cell-clearing pathways challenged the traditional concept of autophagy and UPS as independent systems. In fact, autophagy and UPS are simultaneously affected in a variety of CNS disorders where synaptic and inflammatory/immune alterations concur. In this review, we discuss the role of autophagy and UPS in bridging synaptic plasticity with neuro-immunity, while posing a special emphasis on their interactions, which may be key to defining the role of immunity in synaptic plasticity in health and disease.

## 1. Introduction

In recent years, unexpected connections have emerged between synaptic transmission, inflammatory/immune mediators, and brain (patho)-physiology [1,2,3]. In fact, the prevailing dogma that portrayed the nervous and immune system as two independent entities has been progressively replaced by new levels of functional connections and commonalities [4,5,6]. This interconnection rose up to a level that involves synaptic plasticity concerning both its molecular mechanisms and the clinical outcomes related to behavioral abnormalities [7,8]. Synaptic plasticity refers to those activity-dependent changes in the strength or efficacy of synaptic transmission, which occur continuously upon exposure to either positive or negative stimuli, such as learning, exercise, stress, or substance abuse, as well as the subsequent mood conditions [8]. Modifications of the neural circuits entail a variety of cellular and molecular events, encompassing neurotransmitter release; ionic activity; and metabolic, epigenetic, and transcriptional changes, which converge to shape the neuronal proteome and phenotype in an attempt to restore homeostasis [9,10,11]. The ability to re-establish and/or sustain baseline brain functions depends on a plethora of synchronized activities, which indeed involve both neuronal- and immune-related mechanisms. In this scenario, neurotransmitters and immune-related molecules adopt a common language to fine-tune brain functions [12,13,14,15]. In fact, classic immune molecules, including cytokines, major histocompatibility complex (MHC) molecules, and T-cells, are deeply involved in central nervous system (CNS) plasticity, while CNS factors, mostly neurotransmitters encompassing dopamine (DA) and glutamate (GLUT), actively participate in shaping immune functions [14]. Neuro-immune surveillance is a critical component for brain function, as circulating T-cells that recognize CNS antigens (Ags) are key in supporting the brain’s plasticity, both in health and disease [8]. The functional anatomy from which the molecular interplay between the immune system and brain matter stems, was recently identified at the level of lymphatic pathways operating in the perivascular (also known as “glymphatic”) and dural meningeal spaces [16,17,18]. Lymphatic flows foster the drainage of the brain interstitial fluid into the cerebrospinal fluid, and then back again into the bloodstream, or even directly into the secondary lymphoid organs. Functionally, this translates into a clearance of potentially threatening interstitial solutes and the drainage of CNS-derived Ag peptides to the deep cervical lymph-nodes to be captured and processed by antigen presenting cells (APCs) [19,20]. Within this context, synaptic plasticity, apart from being modulated by classic CNS molecules, is strongly affected by the immune system. This is not surprising, given the common molecular pathways that operate at the cross-road between the nervous- and immune-system. In fact, just like what is happening for the key proteins involved in neurotransmitter release [21,22], Ag processing within APCs is carried out by the two major cell-clearing machineries, ubiquitin proteasome (UPS) and autophagy [23,24,25]. In detail, UPS and autophagy operate both in the CNS and immune system, to ensure protein turnover and homeostasis. In the CNS, UPS- and autophagy-dependent protein degradation is seminal to protect neurons from potentially harmful proteins, and to modulate neurotransmitter release and synaptic plasticity [21,26,27,28]. Similarly, in the immune system, UPS and autophagy cleave endogenously- and exogenously-derived proteins to produce Ag peptides, which bind to MHC molecules class I and II [23,24,25,29]. Indeed, these pathways converge when the CNS components are cleared by immunocompetent mechanisms [24,29]. Thus, CNS-derived Ags bound to MHC-I and –II may be exposed on the plasma membrane of APCs, for presentation to CD8+ and CD4+ T-lymphocytes, respectively [29,30]. The associative binding of MHC molecules with T-cells receptors (TCR), coupled with co-stimulatory signals and the presentation of CNS-derived Ags, fosters the activation of naïve T-cells in the periphery, while mounting CNS-directed adaptive immune responses, which may produce either beneficial or detrimental effects already pertaining to the field of CNS plasticity [2,14,31,32,33]. Still, at anatomical level, the sympathetic innervation of both primary and secondary lymphoid organs provides a means of functional connection between the immune- and nervous-system [34]. In fact, catecholamine, and mostly DA released from sympathetic nerve terminals, is an active regulator of the metabolism, fate, and activity of naïve CD4+ and CD8+ T-cells [35,36]. This is achieved through the binding of DA to its cognate receptors and transporters, which are abundantly expressed on lymphoid cells. Likewise, the GLUT released in the bloodstream or within the CNS modulates T-cells activity, through binding to its cognate receptors, which are expressed on T-cells [2,37]. In this way, neurotransmitters and CNS-derived Ag presentation synergize to define the pool of immunocompetent cells that travel back and forth between the brain and periphery, to guarantee neuro–immune surveillance and synaptic plasticity. Antigen presentation and immune responses may also occur directly in the brain upon interactions between CNS circulating T-cells and glia, or even neurons [38,39,40,41,42]. Unexpectedly, recent studies showed that naïve T-cells are able to cross CNS barriers and infiltrate the brain parenchyma [38,39,40,41,42,43,44,45,46,47]. This is magnified during pro-inflammatory conditions when the glia and even neurons operate as competent APCs, as they become able to process and present Ags via MHC molecules [39,47,48]. At the same time, T-cells possess all of the machinery that is necessary for releasing and responding to neurotransmitters, just like neurons and glia do [35,37]. The existence of such a bi-directional dialogue between nerve and immune cells has now challenged the classical dichotomy between inflammatory and degenerative disorders of the CNS. In fact, defective or inappropriate communication between the immune and nervous system gives rise to a chain of events, where inflammatory/immune and synaptic alterations intermingle to produce CNS disorders, encompassing neuro-developmental, neurodegenerative, and auto-immune diseases [2,12,13,20,49]. When searching for a common pathway bridging neuro–immune and synaptic dysregulations, UPS and autophagy machineries take center stage. The dysregulations of both UPS and autophagy characterize a plethora of CNS disorders, where synaptic and neuro-inflammatory/immune alterations co-exist, such as Parkinson’s, Alzheimer’s, and Huntingtin’s diseases (PD, AD, and HD); epilepsy; ischemia; brain tumors; multiple sclerosis (MS), and psychiatric and substance-abuse disorders [6,21,50,51,52,53,54,55,56,57,58,59,60,61,62,63,64,65,66,67,68,69,70,71,72,73,74,75,76,77]. The reason for such a common dysregulation of UPS and autophagy in etiologically different CNS disorders is rooted in their pleiotropic catalytic functions, which are seminal for both synaptic plasticity and neuro-immunity [30,78,79,80,81,82,83,84,85,86,87]. Despite being traditionally considered as independent systems, recent evidence has unraveled a functional cross-talk between UPS and autophagy, which occurs at both biochemical and morphological levels [73,88,89,90]. Thus, it is not surprising that autophagy and UPS share most of their substrates and functions, and they operate dynamically and coordinately in both nerve and immune cells so as to modulate neurotransmission, oxidative/inflammatory stress response, and immunity [91,92,93,94]. This is accomplished through the degradation and turnover of proteins, including those involved in endocytic and secretory pathways, transcription factors, and oxidized and/or immunogenic proteins. The present review aims to analyze those molecular interactions that are related to both UPS and autophagy, and that enable neurons and immune cells to surveil synaptic and neuro–immune activity. Apart from being well known triggers of synaptic plasticity, environmental agents such as pathogens, inflammatory cytokines, free radicals, and abnormal neurotransmitter release can profoundly affect cell-clearing systems [51,52,94,95,96,97,98,99,100]. As a proof of concept, when a dysregulation of cell-clearing systems occurs, the altered communication between the nervous and immune cells translates into maladaptive plasticity, which may underlie behavioral alterations. Given the variety of specific regulatory signals and molecules involved in the interplay between UPS and autophagy, a better understanding of their interactions is key in order to define the role of immunity in synaptic plasticity in health and disease.

## 2. Cell Clearing Systems: Tracing the Path of the Interplay between Proteasome and Autophagy 

Autophagy and UPS ensure eukaryotic cell proteostasis by clearing unfolded, misfolded, oxidized, or disordered proteins, so as to prevent their accumulation, aggregation, and spreading [60,101,102,103,104,105,106]. Besides being seminal in extreme cell conditions when cell survival is jeopardized, autophagy and UPS activities operate in baseline conditions in order to keep the turnover of proteins that naturally occur within a living cell steady. In fact, as actors of protein degradation, autophagy and UPS regulate most cell functions encompassing cell cycle and division, cell differentiation and development, endo- and exo-cytosis, and, specifically, synaptic strength and Ag processing [6,21,22,25,26,27,107,108,109,110,111]. Autophagy initiates with the formation of double-layered membrane vacuoles, named phagophores. The maturation and sealing of the phagophore leads to the formation of the autophagosome, which stains for autophagy markers such as beclin-1 (the orthologue of yeast Atg6) and LC3 (Atg8) [112,113]. The autophagosome shuttles a variety of substrates, including ubiquitinated proteins and whole organelles (e.g., mitochondria, endoplasmic reticula, ribosomes, and synaptic vesicles) to the lysosomal compartment, which is gifted with a rich enzymatic activity. The merging of the autophagosome with endosomes and lysosomes generates the catalytic organelle autophagolysosome, where the degradation and recycling of “in bulk” sequestered cytosolic cargoes occurs [112,113]. Protein tagging with ubiquitin chains, which is carried out by the UPS system, represents a sorting signal for either UPS- or autophagy-dependent protein degradation [114]. Protein ubiquitination is an ATP-dependent process that is accomplished by three enzymes, namely ubiquitin-activating (E1), ubiquitin-conjugating (E2), and ubiquitin-ligase (E3). Several proteins operate at the cross-road between UPS and autophagy, to regulate the sorting and shuttling of ubiquitinated substrates towards either system. Among these proteins, which indeed constitute a much longer list, three are worth mentioning, namely (i) Parkin, (ii) histone deacetylase 6 (HDAC6), and (iii) Sequestosome-1 (SQSTM1)/p62. 

(i) Parkin is an ubiquitin-ligase enzyme (E3 ligase), which mediates protein polyubiquitination and serves as a signal for targeting misfolded proteins to the aggresome, where autophagy is recruited [115]. Parkin-dependent ubiquitination triggers the removal of the pro-apoptotic proteins BAX and BCL-2 by either UPS or autophagy, and it is seminal to induce mitophagy, that is, mitochondria-specific autophagy [116]. After ubiquitin linkage, Parkin also induces the coupling of target proteins with dynein motor complexes via the adaptor protein HDAC6 in order to facilitate their transport to the aggresome, where autophagy is recruited; 

(ii) HDAC6 is a microtubule-associated histone deacetylase, which shuttles polyubiquitinated substrates along the microtubules for autophagosomal engulfment, while fostering lysosomes transport to the site of autophagy occurrence. In detail, HDAC6 binds the polyubiquitin chains [117,118] or even C-terminal regions of free ubiquitin [119] via a C-terminal zinc finger-containing domain (called BUZ domain). Then, HDAC6 binds to the microtubule-associated dynein motors to shuttle the polyubiquinated proteins to the aggresomes, while fostering the recruitment of autophagy to the aggresomes [120,121]. Again, HDAC6 participates in the fusion of autophagosomes with lysosomes for final autophagy degradation [122,123]. Remarkably, HDAC6 activity is essential for autophagy, to compensate for protein degradation and rescue cell survival when UPS is impaired [124], thus providing a functional link between autophagy and UPS. 

(iii) SQSTM1/p62 is a ubiquitin-binding scaffold protein that links ubiquitinated proteins to autophagy machinery in order to enable their degradation [125]. This occurs through a direct interaction between SQSTM1/p62 and ubiquitinated proteins via a C-terminal UBA domain, and their subsequent binding to autophagy proteins such as LC3 and GABARAP family proteins. As p62 is itself degraded by autophagy, it is widely used as a marker of autophagy flux [126].

**Figure 1:**
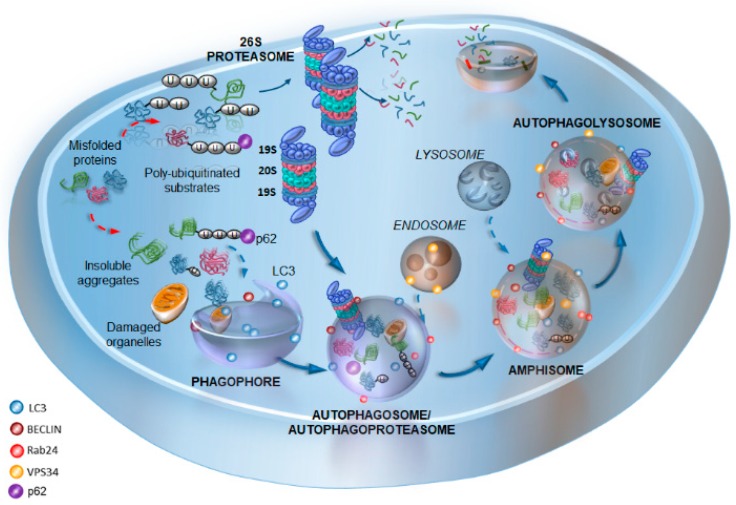
A schematic overview of the cell-clearing systems, with a focus on the interplay between autophagy and proteasome. The cartoon offers a rough schematization of the autophagy pathway, starting from the phagophore biogenesis staining for LC3 and Beclin1, which engulfs cytoplasmic portions containing insoluble aggregates, ubiquitinated substrates, and damaged organelles. The phagophore membrane seals to from the autophagosome, which then fuses with the late endosomes to generate the amphisome. This latter fuses with lysosomes to generate the autophagosome (staining for LC3, Beclin1, Rab24, VPS34, and p62) where cargo degradation occurs. At the same time, ubiquitinated substrates can be degraded by the 26S ubiquitin proteasome (UPS), which is formed by the regulatory subunits 19s and the catalytic subunits 20s. Signals such as p62 contribute to sort ubiquitinated proteins for either UPS or autophagy degradation. At the same time, p62 may serve as a signal to promote the merging of UPS and autophagy into a single organelle, “autophagoproteasome”, where potentiated cell-clearance may take place. Alternatively, the autophagy-dependent degradation of inactive UPS subunits may occur within this compartment, a phenomenon that is named “proteophagy”. Dotted arrows indicate the formation of insoluble aggregates from misfolded proteins and their ubiquitination (red), the shuttling of substrates to the phagophore (blue), and the fusion of endosomes and lysosomes with autophagy vacuoles (blue). Solid blue arrows indicate the progression of the autophagy machinery, the shuttling of ubiquitinated substrates to the UPS, and the shuttling of the UPS within autophagosomes.

Once tagged with ubiquitin, proteins are recognized by autophagy and/or the proteasome 26S (P26S) multimeric complex, which is formed by a catalytic core (P20S) and two regulatory subunits (P19S, also known as PA700) capping the ends of P20S [127]. P19S binds the poly-ubiquitin chain and cleaves it from the substrate. In this way, the unfolded substrate enters the P20S to be degraded by the β1, β2, and β5 catalytic subunits of the P20S, which own chymotrypsin-like, trypsin-like, and caspase-like activity, respectively. Despite being traditionally considered as cytosolic catalytic machinery, UPS also associates with vesicular organelles, including precursor synaptic vesicles (SVs), Golgi-derived vesicles, mitochondria, and lysosomes [128]. Far from being a mere phenomenon of morphological co-localization, the association of UPS with vesicular structures probably underlies a sophisticated functional cooperation. In fact, vacuolar organelles may serve as a ferryboat to shuttle UPS in different cell-compartments, while the UPS handles the turnover of vesicle-associated proteins. This is in line with the recent studies characterizing a novel organelle named “autophagoproteasome”, where the autophagy and UPS markers co-localize [73,88] (Figure 1). The formation of this specific vacuolar compartment is hindered by the administration of the neurotoxic abused drug methamphetamine (meth), while its rescue via the inhibition of the mammalian target of rapamycin (mTOR) correlates with cell protection and survival [73]. This is line with studies indicating the mTOR pathway as a common modulator of both UPS- and autophagy-dependent protein degradation [89]. These findings configure mTOR inhibition as a potential strategy to synergistically enhance autophagy and UPS-dependent protein degradation. mTOR is a ubiquitously expressed serine-threonine kinase, which senses and integrates several environmental and intracellular cues to orchestrate major processes, such as cell growth and metabolism [55,75,129]. mTOR has been widely implicated in synaptic plasticity, inflammation, and immunity, although this was merely related to the role in protein synthesis. In the last decades, mTOR has been posed at the center stage on a variety of cell functions, mostly related to autophagy and UPS. The emerging mechanisms linking mTOR with autophagy and UPS unravel a close interdependency between the cell-clearing systems. In detail, the duration and amplitude of the autophagy response depends on the stability of the serine/threonine kinase ULK1/Atg1, which, in turn, is coordinately regulated by UPS and mTOR [130]. ULK1 acts at multiple steps of autophagy initiation and response, in part by phosphorylating autophagy proteins, including Atg13, Beclin 1, and Atg9 [131]. mTOR activation inhibits ULK1 kinase activity (and thus autophagy initiation) via phosphorylation, and also coordinates ULK1 de novo protein synthesis [130,132]. In this context, UPS behaves as a sentinel in sensing and regulating mTOR/ULK1-dependent autophagy. In fact, during the early stages of autophagy, UPS mediates the K63-linked polyubiquitination of ULK1 via the AMBRA1–TRAF6 (E3 ligase) complex to maintain its stability, self-association, and kinase activity [133]. Conversely, during prolonged nutrient starvation, UPS targets ULK1 for degradation, following Cullin/KLHL20-dependent K48-linked polyubiquitination, thus providing a feedback control of the autophagy response [134]. In turn, autophagy may control UPS efficacy and activity through the degradation of inactive UPS subunits, which are shuttled to autophagosomes, a phenomenon known as “proteophagy” [135,136,137]. This may explain the intriguing effects that are observed on autophagy upon UPS inhibition, and vice versa, while remarking on the importance of autophagy-UPS cross-talk in cell homeostasis. In fact, the inhibition of either autophagy or UPS alone may produce detrimental effects to cell survival [138,139,140,141,142,143], which are bound to impaired protein turnover by both cell-clearing systems. For instance, autophagy inhibition leads to the accumulation of ubiquitinated substrates by affecting UPS either upstream, or at the level of its catalytic activity [144,145]. Conversely, UPS inhibition may induce an enhancement of autophagy as an early compensatory response to cope with protein overload and grant cell-survival [146,147,148]. Such an effect turns out to be only transitory, as UPS dysfunction at later stages impedes mitophagy and decreases the levels of essential autophagy proteins, such as Atg9 and LC3B [93]. This is not surprising, as UPS is essential for endo–lysosome membrane fusion [149,150], which, in turn, is involved in the late steps of autophagy. In fact, UPS modulates the activity of Rab GTPases (GTP-bound Ras proteins in the brain), which are involved in all cell-trafficking mechanisms, including autophagy-dependent endocytosis and autophagy membrane fusion [150,151,152,153]. These findings indicate that the synergistic and compensatory functional interplay between autophagy and UPS needs to be taken into account in experimental approaches modulating either systems alone. On the one hand, this may lead to confounding outcomes when assessing the effects of autophagy and UPS alone; on the other, it calls for investigating the potential strategies that can simultaneously rescue the defects of autophagy and UPS. In keeping with this, it is worth of mentioning that UPS exists as two alternative isoforms, the standard 26S proteasome and the immuno-proteasome (SP and IP, respectively). It is remarkable that the mTOR pathway also modulates the switch between these alternative subtypes of UPS, which evolution has preserved in order to optimize different tasks according to specific cell demands [154,155,156,157]. In fact, SP is ubiquitously expressed in all eukaryotic cells, and it is generally enhanced by mTOR inhibition, while IP is an alternative, cytokine-inducible form that is downregulated by mTOR inhibition. Despite overlapping in structure and functions, these alternative UPS isoforms differ in catalytic subunits and substrate specificity [83,84,85,101,102,103]. In fact, the IP operates constitutively in all immune-related cells, including professional APCs (e.g., dendritic cells—DC) and lymphocytes, and thus, it is mostly involved in potentiating innate and adaptive immunity [158]. 

Remarkably, persistent oxidative/inflammatory stress may concomitantly affect autophagy flux and IP–SP switch, either in the immune periphery or within the CNS. This is expected to alter the clearing capacity and/or substrate specificity of the cell-clearing systems, while triggering a cascade of molecular events that synergize to produce synaptic dysfunctions/toxicity, along with a loss of auto-immune tolerance up to the development of CNS-directed inflammatory and auto-immune reactions. In the present manuscript, we discuss the role of autophagy, SP, and IP at the level of classic neuronal and immunological synapses, while posing a special emphasis on their effects at the level of hybrid junctions, which establish “neuro-immunological synapses” between immune and nerve cells. This is critical to comprehend those autophagy and UPS-dependent mechanisms that finely tune T-cells populations that migrate to the CNS. Again, the role of autophagy and UPS is seminal to disclose those molecular events, which induce neurons and glia to behave as competent APCs, and, as such, become possible targets for auto-immune damage. 

## 3. Autophagy and Proteasome Tune Synaptic Plasticity by Modulating Neurotransmission and Immunity

Autophagy and SP are constitutively expressed in neurons either in the cell body, nucleus, or synapses, where they modulate synaptic plasticity by surveilling oxidative stress, gene transcription, and neurotransmitter release [22,159,160,161,162,163,164,165,166,167,168,169,170,171]. Autophagy and SP operate at various sub-cellular levels in both pre- and post-synaptic sites, and in detail, they are the following:

(i) intersect with secretory pathways to modulate SV trafficking, as well as the size and number of SV pools [21,128,161,172,173];

(ii) degrade protein isoforms and presynaptic chaperone proteins such as alpha-synuclein, beta amyloid, and tau [62,65,108,174,175], which, when altered in either amount or conformation, can drive synaptic dysfunctions [176,177,178,179];

(iii) modulate the rate and duration of neurotransmitter release (including DA and GLUT) by degrading whole SVs (in the case of autophagy), as well as soluble Nsf attachment protein receptor (SNARE) and accessory proteins, which are involved in SV exo-/endo-cytosis [21,22,152,161,162,166,167,168,180,181,182,183];

(iv) foster the internalization and degradation of DA and GLUT receptors, which are coupled with downstream intracellular cascades driving metabolic, transcriptional, and epigenetic changes within neurons [11,98,184,185,186,187]. As such, autophagy and SP are deeply involved in those mechanisms driving synaptic plasticity, such as long term-potentiation and -depression, which are directly related to neuronal and behavioral phenotypes. In fact, the inhibition of either SP or autophagy in experimental models produces profound alterations in neurotransmitter release and the expression of neurotransmitter receptors [31,161,173,181,182,183]. Reiterated stimuli that alter neurotransmitter activity are seminal to induce maladaptive changes in synaptic strength and connectivity, which translate into long-lasting psychomotor changes. In the last decades, experimental evidence has accumulated, suggesting that early synaptic alterations may represent a major event fostering neuronal degeneration [2,188,189]. This is best exemplified by the mechanisms of action of abused drugs such as meth, which produces psychiatric alterations including addiction and psychoses, and even neurotoxicity affecting the DA terminals, DA cell bodies, and post-synaptic neurons of the DA circuitry within the striatum, iso-cortex, and limbic brain areas [11,190,191,192]. This occurs through the joined contribution of epigenetic events and protein alterations (oxidation, aggregation, and spreading) arising from abnormal DA release, the abnormal pulsatile stimulation of DA receptors, and also the increased responsiveness of neurons to GLUT and GLUT exitotoxicity. In fact, abnormal levels of DA and abnormal stimulation of DA receptors play a key role in GLUT excitotoxicity, which stands for the over-activation of specific types of GLUT receptors, resulting in neuronal death, tissue damage, and loss of brain function, as it occurs both during meth toxicity and in various neurological diseases [2,193].

Both autophagy and UPS are severely affected by meth administration [70,71,72,73], while the mTOR inhibitor rapamycin prevents both the behavioral and neurotoxic effects of meth by rescuing autophagy and UPS [73,194]. This is in line with several studies showing that the genetic or pharmacological occlusion of autophagy and UPS leads to the accumulation of ubiquitinated protein-aggregates and recapitulates neurodegeneration [138,139,140,141,142,143,195]. As a support to these findings, SP and autophagy dysfunctions occur in human brain disorders characterized by early synaptic dysfunctions, which precede protein aggregation [6,21,75,196,197,198]. On the other hand, mTOR inhibition, which is supposed to restore both autophagy and UPS activity, ameliorates early psychomotor and cognitive behavioral alterations by recuing neurotransmission defects and by restoring proteostasis in a variety of CNS disorders, both in humans and experimental models [75,175,194,199,200,201,202].

### 3.1. Autophagy- and Proteasome-Dependent Neurotransmission Linking Immune-Cells’ Activity and Synaptic Plasticity

**Figure 2:**
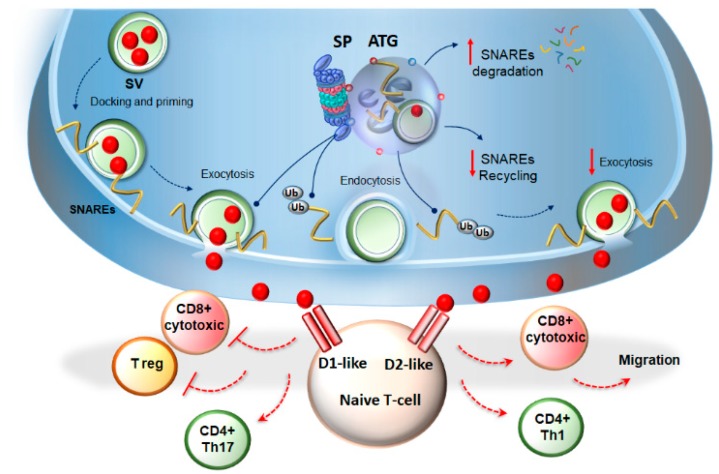
Autophagy and proteasome modulate immune activity by surveilling dopamine (DA) release. The standard proteasome (SP) and autophagy blunt DA release by degrading entire synaptic vesicles (SVs), as well as soluble Nsf attachment protein receptor (SNARE)- and SNARE associated-proteins, which foster synaptic vesicle exocytosis. In fact, they both prevent the rapid recycling of SV proteins back to the plasma membrane, which would otherwise lead to a further round of exocytosis. In this way, SP- and autophagy-dependent amount and duration of DA released at the level of the neuro-immunological synapse surveils the stimulation of DA receptors expressed on T-cells. This is seminal to modulate the differentiation of T-cells toward cytotoxic-, regulatory-, or helper-T-cells, as well as T-cell migration in periphery. For instance, the abnormal stimulation of D1-like receptors increases cyclic adenosine monophosphate (cAMP) levels to inhibit cytotoxic CD8+ cells; it impairs the differentiation and activity of T-regulatory cells, while inducing polarization of naive CD4+ cells toward the Th17 phenotype. On the other hand, the stimulation of D2-like receptors induces the differentiation of CD8+ cells into cytotoxic T-lymphocytes, induces the polarization of naive CD4+ cells toward the Th1 phenotype, and controls T-cell migration and adhesion. Dotted blue arrows indicate the progression of the SV cycle. Solid blue arrows indicate the targeting and shuttling of SNARE proteins to the UPS and autophagy. Solid red arrows indicate the increase/decrease in SNAREs degradation/recycling and exocytosis rate. Dotted red lines indicate the induction (arrows) or inhibition (lines) of naïve T-cells differentiation towards various phenotypes following abnormal stimulation of DA receptors.

As modulators of neurotransmitter release, autophagy and SP also modulate CNS-directed immune responses by operating at the level of the neuro-immunological synapse, which may be established between the sympathetic nerve terminals and T-cells within lymphoid organs [12]. Remarkably, both SP and autophagy modulate the release of DA [161,162,181,182,183], which besides being crucial for brain functions such as movement, cognition, attention, memory, and reward [203], also orchestrates the differentiation, maturation, selection, trafficking, and migration of T-lymphocytes [34,35,36,204,205,206]. In fact, T-cells express G-coupled D1-like (D1 and D5) and D2-like (D2, D3, and D4) DA receptors, and just like it occurs for the neurons, the magnitude and duration of the DA release is key to trigger the specific metabolic and intracellular cascades, switching T-cells phenotype and function [35,36,206]. As thoroughly revised elsewhere, depending on the DA concentration and the pattern of stimulation of specific DA receptors, naïve T-cells may be induced to differentiate into either memory, regulatory, or effector cells, including CD4+ T helper (Th) 1, 2, or 17, and CD8+ cytotoxic T-lymphocyte (CTL) phenotype [34,35,36,206]. In this context, the autophagy- and SP-dependent surveillance of the DA release at the level of the neuro–immunological synapse is expected to guarantee the physiological stimulation of the DA-receptors placed on the T-cells, and control the neuro–immune activity (Figure 2). The circulation of T-lymphocytes in the brain occurs physiologically, since the early development, and persists during adulthood, to guarantee synaptic plasticity [8,207,208]. For instance, both CD4+ and CD8+ T-cells are essential for spinogenesis and GLUT synaptic function in the hippocampus [209]. In addition, CD8+ T cells regulate the hippocampal volume by promoting neurogenesis [210]. Intriguingly, peripheral and brain-infiltrating T-cells, besides regulating GLUT synaptic transmission and plasticity, are regulated themselves by GLUT [2,37]. Both autophagy and UPS modulate, and are in turn modulated by GLUT transmission [95,211,212,213]. GLUT is a major excitatory neurotransmitter, which besides being critical for the brain’s development and function, participates in tuning the T-cells activity. In fact, ionotropic and metabotropic GLUT receptors are differently expressed among resting and activated T-cells, as well as in different T-cells subtypes [2,37]. At low physiological concentrations, GLUT promotes T-cell adhesion, migration, proliferation, and protection of activated T-cells from Ag-induced apoptotic cell death. Yet, depending on the abnormalities concerning either the GLUT concentration, stimulation of specific GLUT receptors, or the presence of other converging stimuli (such as inflammatory cytokines or other neurotransmitters), GLUT may profoundly affect T-cells activity, thus playing an active role in immune diseases [2,37]. Remarkably, brain infiltrating T-cells were shown to respond to GLUT by activating a neuroprotective pathway, thus providing a potential feedback regulatory mechanism to limit GLUT excitotoxic damage in the CNS [214]. A loss of GLUT-mediated responsiveness of T-cells has been described in MS [215]. Furthermore, various alterations in CNS-circulating T-lymphocyte populations are described in both classic and autoimmune degenerative disorders, such as PD, AD, and MS [216]. Emerging evidence also indicates an association between early inflammatory mechanisms underlying neurodegeneration, and synaptic alterations involving abnormal levels of DA and/or GLUT, as well as the deregulation of their receptors on T-cells [2,37,217,218,219]. In line with this, specific modulators of DA and/or GLUT activity, may have beneficial effects, not only in classic neurodegenerative diseases, but also in auto-immune CNS disorders such as MS [2,35,217]. 

Besides the effects in T-cells within the lymphoid organs, DA and GLUT release may also modulate the activity of immune cells, including glia and T-cells, directly in the CNS. In line with this, a number of studies pointed to the unexpected ability of naïve CD4+ and CD8+ T-cells to infiltrate the brain parenchyma [38,39,40,41,42,43,44,45,46,47]. This occurs mostly during pro-inflammatory conditions, which enhance naïve T-cell recruitment in the CNS, while fostering T-cells activation and phenotypic commitment once they encounter activated glial cells exposing MHC-bound Ags. Thus, the effects of DA and GLUT on brain-infiltrating naïve T-cells may synergize with local Ag presentation in order to dictate the activation or suppression of T-cells directly in the CNS. However, as a general consensus view, only peripherally activated T-cells can migrate into the brain. If they encounter a CNS-resident APCs exposing the cognate Ag, T-cells become re-activated and recruit their effector machineries to produce cytotoxicity or cytokine release. In this scenario, the effects of DA and GLUT focus mostly on the glial cells, which behave as CNS-resident immune cells. Inflammatory cytokines (such as those released by brain infiltrating T-cells) synergize with neurotransmitters to induce the activation of the glial cells, which encompass the morphological changes, increased proliferation rate, and ability to operate as APCs [9,220,221]. Nonetheless, because of an intimate functional association with the synapses, the glia is deeply involved in synaptic plasticity [9]. Experimental studies suggest that activated microglia are responsible for the synaptic alterations observed in a variety of neurological disorders [2,222]. Both microglia and astrocytes express many different neurotransmitter receptors (including DA and GLUT receptors), which, when stimulated, foster the release of soluble factors acting, in turn, on neurons to alter neurotransmitter release, neurotransmitter receptor activation, and synaptic efficacy [2,9]. Besides neurotransmitters such as DA and GLUT, these include mediators of constitutive immunity such as tumor necrosis factor alpha (TNFα), interferon gamma (IFNγ), and interleukin 1 beta (IL-1β); pentraxins; and growth factors such as brain-derived neurotrophic factor (BDNF), which altogether influence synaptic activity, mostly by enhancing the long-term potentiation of excitatory transmission [2,9,77,81,223]. Once again, in this context, autophagy and UPS configure as actors in the communication between T-cells, glia, and neurons, as they (i) surveil neurotransmitter release, which is important for glial, lymphocytic, and neuronal activity; (ii) determine the metabolism and activity of the glia and lymphocytes and the subsequent production and release of soluble factors (paragraph 3.2); and (iii) process Ag peptides, which are presented to T-cells by DCs, glial cells, or neurons (paragraph 3.3). 

### 3.2. Cell Clearing System in the Metabolism and Fate of Immune Cells

Similar to what occurs in the neurons, the activity of immune cells largely depends on UPS and autophagy. The multitude of metabolic changes that occur upon glial and T-cells activation is tightly intermingled with autophagy and UPS activity. As detailed in the previous paragraph, both UPS and autophagy regulate DA and GLUT release, and are also involved in the turnover of DA and GLUT receptors, thus influencing the metabolic cascades, which participate in T-cells and glia activation. Moreover, both autophagy and UPS modulate the turnover of inflammatory-related transcription factors such as nuclear factor k beta (NF-κB), which, in turn, fosters the production of cytokines by glia or T-cells [25,224,225,226]. Within T-cells, autophagy and UPS directly govern the metabolic cascades, which dictate T-cells differentiation, function, and activity [6,158,226]. Again, such an overlapping task may be bound to mTOR activity, which is deeply involved in T-cell metabolism [227]. Moreover, the co-existence of UPS and autophagy degradation pathways enables APCs (including peripheral DCs and glia) to present either endogenous or exogenous Ag peptides, which is key for determining the T-cells state [30]. While autophagy operates constitutively in all immune cells, the UPS exists mostly as IP, which is an alternative, cytokine-inducible isoform of the SP possessing enhanced chymotrypsin-like activity and peculiar structural features, compared with SP [228,229,230,231,232]. In fact, within IP, β1, β2, and β5 subunits of the SP-20S catalytic core are replaced with β1i, β2i, and β5i. Among these, β1i possesses a chymotrypsin-like activity contrarily to the standard counterpart possessing a caspase-like activity. Thus, compared with SP, IP produces, more efficiently, Ag peptides with C-terminal hydrophobic amino acids, which are suitable for binding the groove of MHC class I molecules [228,229,230,231,232]. The major task of IP is to process either endogenous or exogenous proteins, and generate Ag peptides, which are first complexed to MHC-I in the endoplasmic reticulum, and then exposed on the plasma membrane of APCs for either direct or cross-presentation to CD8+ T-lymphocytes. Thus, the generation of defined T-cell epitopes and the expression of MHC-I molecules largely depend on the IP activity [6,35,228,229,230,231,232]. Similar to IP, autophagy is key in adaptive immunity, though it is mostly implicated in the MHC-II restricted presentation of exogenously-derived Ag to CD4+ T cells [23,30]. Nonetheless, a few reports demonstrated that autophagy can also process and load endogenous (viral) peptides to MHC-I [233]. Remarkably, autophagy is implicated in MHC class I molecules internalization and degradation, thus influencing MHC-I stability at the plasma membrane of APCs, and subsequent CD8+ T-cell responses [234,235]. In fact, autophagy inactivation within APCs occludes the surface internalization of MHC-I molecules, leading to an increased Ag presentation and enhanced CD8+ T cell responses against viral peptides, both in vitro and in vivo [233]. Thus, autophagy fosters MHC II-restricted Ag presentation, while controlling MHC-I expression [233,234,235]. Autophagy also provides an alternative pathway to the direct IP- and MHC-I-dependent Ag presentation pathway [236]. In this case, Ags normally targeted to autophagy and exposed by MHC-II can also be loaded to MHC-I in recycling endosomes, which is seminal to trigger adaptive immune response upon viral infections. In the immune periphery, autophagy- and UPS-dependent Ag processing is also seminal for T-cells thymic selection. In the thymus, specialized forms of IP operate together with SP and autophagy to finely-tune T-cell proliferation, along with positive and negative T-cell selection [228,237]. In this way, UPS (SP and IP) and autophagy coordinately guarantee immune-tolerance and define the pool of immunocompetent T-cells, which are released in the bloodstream to reach secondary lymphoid organs, and subsequently, the brain. 

## 4. Autophagy and Proteasome Linking Altered Immunity and Synaptic Plasticity with Neurodegeneration

**Figure 3:**
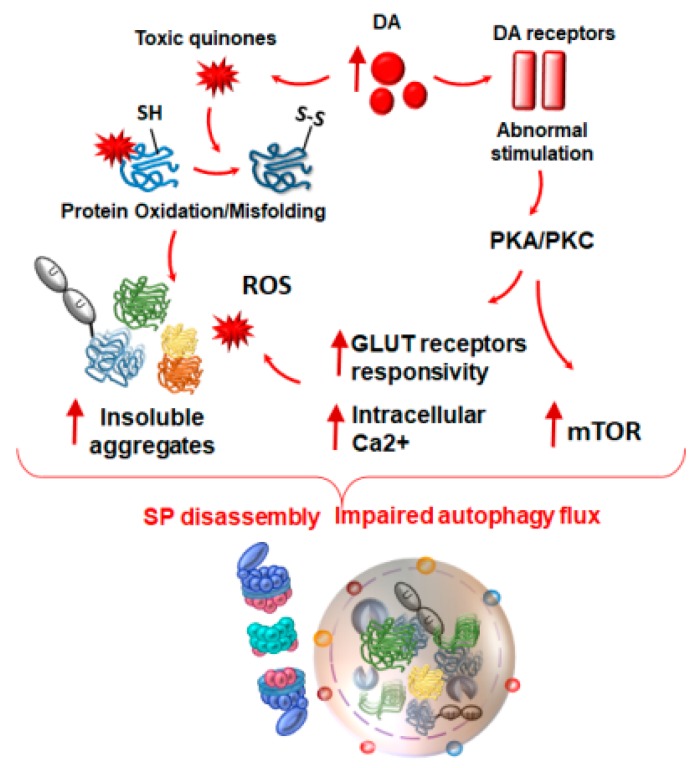
The effects of abnormal DA release and stimulation of DA receptors on proteasome and autophagy. An abnormal amount of intracellular DA may foster the loss of compartmentalized physiological oxidative deamination of DA, which readily undergoes auto-oxidation to produce toxic quinones and highly reactive chemical species such as reactive oxygen species (ROS). In turn, these react with sulfhydryl groups and promote the structural modifications of proteins, lipids, and nucleic acids within the DA axon terminals and surrounding compartments. Structural modifications of proteins translate into the formation of insoluble aggregates overwhelming both the SP and autophagy degradative potential. At the same, the abnormal release of DA produces an abnormal stimulation of post-synaptic DA receptors, which are coupled with intracellular cascades such as protein kinase A and C (PKA/PKC). The non-canonical activation of these cascades promotes the hyper-phosphorylation and activation of glutamate (GLUT) receptors and ion channels, which foster GLUT hyper-responsivity and Ca2+ uptake converging in the increase of oxidative stress. Again, the intracellular cascades placed downstream of the DA receptors (mostly D1-like) converge on activating the mammalian target of rapamycin (mTOR) pathway, thus promoting SP and autophagy downregulation. Red arrows in bold indicate increased levels. Plain red arrows indicate the formation of DA-derived toxic quinones and oxidized/misfolded proteins up to insoluble aggregates, and the progression of the various metabolic cascades that arise from abnormal stimulation of DA receptors.

In neurons and glial cells, autophagy and SP operate constitutively, while the IP is generally induced by the pro-inflammatory cytokines IFNγ and TNFα, and by oxidative stress [85,158,229]. These challenging conditions contribute to disassemble SP for the sake of IP induction, which is likely to cope with the protein overload, as it is endowed with an enhanced catalytic activity [238,239]. Remarkably, the IP cleaves both microbial- and oxidized/aggregated-proteins to produce immunogenic peptides, which are exposed on glial and neuronal MHC-I for presentation to CD8+ T cells. In fact, IP is able to degrade aggregation-prone proteins such as alpha-synuclein and beta amyloid, which are conventionally degraded by SP and autophagy, although some debate still exists concerning the degradation rate and efficacy of IP compared with SP [238,240,241]. In any case, the IP-dependent degradation of aggregation-prone proteins produces Ag peptides, which activate adaptive immunity [6,238]. This provides an oxidation-linked explanation for the baseline activity of UPS in neuro-immune surveillance [85,238]. IP recruitment may serve as a compensatory pro-survival mechanism, allowing cells to quickly expand the peptides repertoire and aid immune defense in a challenged organism. This is supported by the fact that IP also operates in baseline conditions in neurons and glia, which indeed express low amounts of IP and MHC-I, even in the absence of cytokine stimulation [238,242,243]. In line with this, MHC-I-selective expression within the neurons and glia throughout the brain and spinal cord extends well beyond a classic antigen-presenting role. In fact, the MHC-I neuronal expression is key in early neuronal development, axonal regeneration, synaptic plasticity, reward, and memory [243,244,245,246]. Nonetheless, IP induction is a tightly regulated and transient response, as cells must rapidly switch back to SP once the IP function is no longer required [247]. Abnormal IP expression and the subsequent MHC-I-dependent Ag presentation enhances the APC-like behavior of neurons, and, as such, increases their susceptibility to CD8+ auto-immune attack. In fact, a dramatic increase in the amount of IP is bound to an abnormal auto-immune response in a variety of CNS disorders [6]. As recently reviewed, IP is significantly and constantly up-regulated in the glia and neurons, both in patients and experimental models of classic and auto-immune neurodegenerative disorders [6]. Nonetheless, the functional role of IP induction differs between auto-immune compared with classic neurodegenerative disorders. In neurodegenerative disorders such as PD, AD, and HD, the upregulation of IP occurs as a compensatory response to cope with inflammatory conditions that develop during proteinopathy, when SP is downregulated [6,238,248,249]. In fact, general UPS inhibitors targeting both SP and IP produce a detrimental effect, which recapitulates neurodegeneration, while selective IP inhibitors have only limited beneficial effects in the models of neurodegenerative disorders [6]. On the other hand, in auto-immune disorders, including MS and experimental autoimmune encephalomyelitis (EAE), IP inhibitors significantly ameliorate neurological and inflammatory disease scores [6,250]. There is also evidence indicating that a combination of autophagy and IP inhibitors may be an effective strategy against EAE [251]. In keeping with this, a number of studies reported that exposure to cytokines, such as IFN-γ, also up-regulates autophagy to promote the activation of innate and adaptive auto-immunity [252]. In this context, autophagy has been suggested to represent a tolerance-avoidance mechanism, being strongly recruited during CD4+ T-cells activation [253]. Instead, autophagy inhibition induces a long-lasting state of hypo-responsiveness within T-cells [253]. In vivo, autophagy inhibition during Ag priming induces T-cell energy, and decreases the severity of disease in EAE [253]. On the other hand, studies in humans showed that autophagy activity is not increased in neither the peripheral nor brain-circulating CD4+ T cells of MS patients compared with controls, despite having increased Atg5 gene and protein levels [254]. Other studies centered on the role of microglia-related inflammation suggest that autophagy induction via mTOR inhibition contributes to reducing both demyelination and inflammation in EAE [255]. As far as it concerns neurodegenerative disorders, autophagy induction seems to play a beneficial effect in counteracting acute and chronic inflammation [256]. For instance, in an in vitro model of PD, TNF-α was shown to impair autophagy flux in microglia, while fostering microglia polarization towards the pro-inflammatory phenotype M1 [257]. The inhibition of autophagy consistently aggravates M1 polarization induced by TNF-α, and remarkably, autophagy inhibition alone is sufficient to trigger microglia activation toward M1 status, along with producing neurotoxicity [257]. Conversely, the upregulation of autophagy via serum deprivation or pharmacologic activators (rapamycin and resveratrol) promotes microglia polarization toward the M2 phenotype, thus fostering inflammation resolution and preventing neurotoxicity [257]. Again, enhancing autophagy in the microglia in an in vitro model of AD promotes the degradation of the phagocytosed fibrils of amyloid beta, along with restraining the inflammasome activation and pro-inflammatory cytokine release [258]. This is in line with findings indicating that impaired autophagy in microglia associates with synaptic defects, and with the subsequent psychiatric alterations observed in experimental models [76,77]. Again, the disruption of autophagy within neurons occurs following infection-induced microglial activation, which results in neurodegeneration [52]. This is in line with the plethora of studies pointing at autophagy dysfunction in neurodegenerative disorders, such as AD, PD, and HD. In these disorders, a progressive dysfunction of autophagy within the CNS is reminiscent of that reported for SP. In a scenario where the autophagy–UPS interplay appears critical, it is worth of considering some overlapping molecular mechanisms that may operate in various CNS disorders to foster neuro-inflammation and maladaptive synaptic plasticity through IP induction and concomitant SP-autophagy downregulation. Remarkably, autophagy and UPS activities are influenced by the same intracellular cascades that are triggered by the DA receptors expressed on the neurons and glia. In fact, signaling pathways placed downstream to plasma membrane D1-like and D2-like DA receptors converge on the mTORC1 pathway [96], which, in turn, may either suppress or enhance the baseline SP/IP and autophagy activities, depending on the pattern of stimulation of the specific DA receptors. Thus, a feedback loop is established between DA signaling and mTOR-dependent cell-clearing systems in neurons, glia, or even in T-cells. The intrinsic oxidative potential of DA, along with the abnormal stimulation of DA receptors, are primary candidates fostering protein oxidation, inflammation, impairment of autophagy flux, SP disassembly, and the subsequent IP upregulation [48,71,96,97,99,259,260] (Figure 3). This is supported by the effects of exogenously administered DA precursors in enhancing neuronal Ag presentation via MHC-I, and the subsequent activation of CTLs [48], which, in fact, is a major task of the IP. 

**Figure 4:**
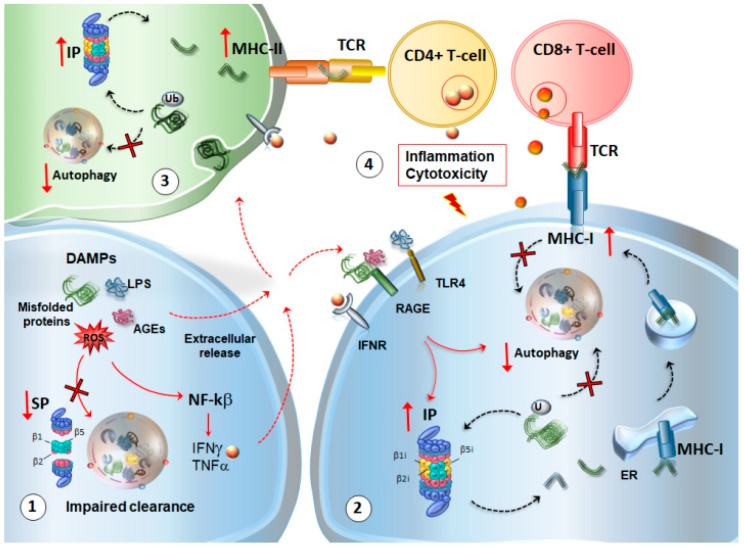
Molecular mechanisms bridging neuro-inflammation, immunoproteasome (IP) induction and autophagy-SP dysfunction in the central nervous system (CNS). (1) As a consequence of autophagy and SP dysregulation, indigested misfolded or oxidized substrates may perpetuate inflammation through the release of danger-associated molecular pattern molecules (DAMPs), such as advanced glycation end-products (AGEs), lipopolysaccharides (LPS), and ROS. While DAMPs activate NF-κB and the inflammasome to release cytokines such as interferon gamma (IFNγ), the spreading of indigested misfolded proteins, AGEs, and free radicals in the extracellular space occurs. (2) All of these factors converge to induce an upregulation of IP within the neighboring cells via autocrine or paracrine mechanisms. DAMPs may also stimulate AGE receptors, IFN receptors, and toll-like 4 receptor (TLR4), to converge on molecular pathways such as mTOR, which, in turn, induce IP upregulation and SP-autophagy downregulation. In this scenario, IP upregulation leads to an overproduction of CNS self-antigens co-expressed with major histocompatibility complex (MHC)-I molecules to prime cytotoxic CD8+ T cell response. At the same time, autophagy cannot efficiently provide for the internalization of MHC-I molecules or the degradation of damaged proteins and organelles. (3) Within glial cells, the same DAMPs and cytokines that foster glial activation may contribute to the impairment of autophagy and SP, while up-regulating IP, which is able to process and cross-present phagocytosed proteins via MHC-II for the activation of CD4+ T cells. (4) In this way, IP upregulation, in an attempt to compensate for SP-autophagy downregulation, may fuel inflammation and auto-immune activation to promote altered synaptic plasticity and neuronal damage. Red arrows in bold indicate decreased/increased levels/activity of SP, IP, autophagy, MHC-I, MHC-II. Plain red arrows indicate intra-cellular signaling cascades. Dotted red arrows indicate the extracellular release of DAMPs, cytokines, and their binding to cognate receptors in neighbor cells or phagocytosis by glial cells. Dotted black arrows indicate the shuttling of substrates towards UPS or autophagy, the formation of Ag peptides deriving from UPS cleavage, and the progression of MHC-I-Ag complex from the ER and endosomes to the plasma membrane. The red frame indicates the final effect produced by CD4+ and CD8+ T-cells activation on neurons and glia.

As a consequence of the autophagy and SP dysregulation, indigested misfolded or oxidized substrates may perpetuate inflammation through the release of danger-associated molecular pattern molecules (DAMPs) (Figure 4). In fact, DAMPs activate NF-κB and the inflammasome to release cytokines such as IFNγ, along with spreading misfolded proteins, advanced glycation end-products (AGEs) and free radicals, which all converge to induce the upregulation of IP within neighboring cells via autocrine or paracrine mechanisms. DAMPs may also stimulate toll-like 4 receptor (TLR4) to impair both SP and autophagy [75]. In this scenario, impaired SP and autophagy can neither digest potentially harmful DAMPs, nor restrain the release of DA and GLUT, which may add on glia activation and the release of pro-inflammatory signals recruiting T-cells within the CNS. In this way, IP upregulation leads to an overproduction of neuronal and glial antigens co-expressed with MHC-I molecules to prime cytotoxic CD8+ T cell response (Figure 4). At the same time, autophagy cannot efficiently provide for the internalization of MHC-I molecules or the degradation of damaged proteins and organelles, which fuels inflammation and immune activation. Thus, alterations of autophagy and UPS may explain why a variety of CNS disorders feature concomitant alterations in neurotransmitter activity, oxidative-inflammatory stress, and inappropriate immune response, which synergize to alter synaptic plasticity and damage neurons.

## 5. Conclusions and Future Directions

The evidence reviewed here suggests that autophagy and UPS are key mediators of synaptic plasticity, being placed at the cross-road between neurotransmission and immune activity. Nonetheless, we are just scratching the surface of the intricate molecular mechanisms that translate autophagy and UPS alterations into specific CNS disorders. Further experimental studies are needed to dissect the correlation between autophagy and UPS status, disease-specificity, and disease-stage. In fact, different effects on autophagy and UPS may occur in auto-immune compared with classic neurodegenerative disorders, because of the different etiologies between these CNS diseases. Moreover, the interdependency between autophagy and UPS may lead to confounding outcomes when assessing the effects of specific compounds, which indeed modulate both systems rather than autophagy or UPS individually. In addition, there are several factors that may contribute to yielding controversial results on autophagy and UPS. One of these may be the biased interpretation of the autophagy or UPS status. In fact, most studies assess autophagy by measuring the amount of LC3B or the number of autophagosomes, which does not necessarily reflect an increased autophagy capacity. Rather, it may reflect a progressive downregulation of autophagy flux due to the impaired fusion of LC3-positive autophagosomes with lysosomes. Again, most studies detect UPS status through antibodies that recognize alpha subunits, which do not allow for distinguishing between the SP/IP ratio. Likewise, measuring the overall UPS catalytic activity does not allow for dissecting whether the contribution derives from SP or IP. In any case, dysregulations of both autophagy and UPS appear as a common signature in a variety of CNS disorders, where synaptic alterations synergize with inflammatory/immune reactions. This calls for further studies aimed at investigating the effects of additional compounds, which can synergistically modulate both UPS (IP and SP) and autophagy, in an attempt to find preventive and/or therapeutic strategies against early synaptic alterations. In keeping with this, mTOR modulators remain, to date, the best candidates for acting on autophagy, IP, and SP. Beyond the gold-standard mTOR inhibitor rapamycin, several phytochemicals, which recently gained increasing interest in CNS disorders due to their adaptogenic, anti-oxidant, and anti-inflammatory effects, act indeed as mTOR modulators. Testing the effects of these compounds on autophagy and SP–IP may disclose a potential correlation between their beneficial effects and cell-clearing pathways.

## Abbreviations

AD	Alzheimer’s disease
Ag	Antigen
APC	Antigen presenting cell
BDNF	Brain derived neurotrophic factor
CNS	Central nervous system
CTL	Cytotoxic T-lymphocyte
DA	Dopamine
DAMPs	Danger-associated molecular pattern molecules
DC	Dendritic cell
EAE	Experimental autoimmune encephalomyelitis
GLUT	Glutamate
HD	Huntingtin’s disease
HDAC6	Histone deacetylase 6
IFNγ	Interferon gamma
IL-1β	Interleukin 1 beta
IP	Immunoproteasome
Meth	Methamphetamine
MHC	Major histocompatibility complex
MS	Multiple sclerosis
mTOR	Mammalian target of rapamycin
NF-κB	Nuclear factor K beta
PD	Parkinson’s disease
Rab GTPase	Gtp bound ras proteins in brain
SNARE	Soluble Nsf attachment protein receptor
SP	Standard proteasome
SQSTM1	Sequestosome-1
SV	Synaptic vesicle
TCR	T-cells receptor
TLR4	Toll-like receptor 4
TNFα	Tumor necrosis factor alpha
UPS	Ubiquitin proteasome
